# Lineage-Specific Disruption of Hematopoiesis by Oxaliplatin: Mechanisms of Erythropoietin Resistance and Immune Suppression

**DOI:** 10.14302/issn.2372-6601.jhor-25-5944

**Published:** 2026-02-16

**Authors:** Leland C. Sudlow, Junwei Du, Kiana Shahverdi, Haiying Zhou, Mikhail Y. Berezin

**Affiliations:** 1 Mallinckrodt Institute of Radiology, Washington University School of Medicine St. Louis, MO 63110, USA; 2 Institute of Materials Science & Engineering Washington University, St. Louis, MO 63130, USA

**Keywords:** Oxaliplatin, Hematopoiesis, Chemotherapy-induced anemia, Erythropoietin resistance, Lineage-specific myelosuppression, Bone marrow transcriptomics, Immune suppression

## Abstract

**Background:**

Oxaliplatin, a widely used chemotherapeutic agent, is associated with hematologic toxicities such as anemia, leukopenia, and thrombocytopenia. Despite their clinical relevance, the molecular mechanisms underlying lineage-specific bone marrow suppression remain poorly understood.

**Methods:**

We administered oxaliplatin to mice over eight weeks and performed RNA-sequencing (RNA integrity >8) on bone marrow alongside peripheral blood analysis and cytokine profiling. Transcriptomic data were analyzed to identify differentially expressed genes (DEGs) and enriched pathways. For that, we applied a thematic Gene Ontology (thematicGO) enrichment method that groups GO terms into biologically meaningful categories, such as hematopoietic lineage disruption, cell cycle arrest, and cytokine signaling.

**Results:**

Oxaliplatin induced broad transcriptional suppression of erythropoiesis and lymphopoiesis, with 3,691 DEGs identified (FDR<0.05, |FC|>1.5). Upregulation of *Cdkn1a* and downregulation of *E2f2* suggest G1/S cell cycle arrest, correlating with repression of key erythroid maturation genes (e.g., *Spta1, Slc4a1, Alas2*) and hemoglobin subunits (*Hba-a1/2, Hbb-bs/t*). Despite a ~3000-fold increase in renal *Epo* expression, bone marrow *Epor* was reduced, indicating erythropoietin resistance. B and T cell markers were also significantly downregulated, signifying a collapse in adaptive immunity. Notably, neutrophil populations were largely spared. Cytokine analysis in plasma revealed a pro-inflammatory shift with elevated TNF-mmunity. NotablGF-d T cell markers were also significantly downregulated

**Conclusions:**

Oxaliplatin induces a lineage-dependent suppression of hematopoiesis, driven by coordinated cell cycle arrest, metabolic stress, and disrupted cytokine signaling. RNA-seq analysis enabled integration of transcriptomic findings into coherent biological themes. These findings provide mechanistic insights into oxaliplatin’s hematologic toxicity linking bone marrow failure (potentially reversible) via interconnected inflammatory and metabolic pathways and may inform therapeutic strategies to minimize or restore myelosuppression in cancer patients.

## Introduction

Oxaliplatin is a third-generation platinum-based chemotherapeutic widely used in the treatment of gastrointestinal malignancies, particularly colorectal cancer. Oxaliplatin is widely used by itself and in combination with other drugs such as FOLFOX-based chemotherapy regimens. While effective against tumors, oxaliplatin causes substantial side effect such as peripheral neuropathy (1, 2) and hematologic toxicity, notably anemia, leukopenia, and thrombocytopenia (3). The hematologic side effects are attributed to direct impacts on hematopoietic stem cell (HSCs) in the bone marrow. Here, we examine systemic blood cell deficits and their molecular correlates in bone marrow post-oxaliplatin exposure to identify transcriptional programs involved in hematopoietic suppression.

According to the FDA-approved labeling (3), oxaliplatin-induced myelosuppression disrupts normal hematopoiesis, leading to decreased production of erythrocytes (anemia, 81% of patients), leukocytes (leukopenia (76%), neutropenia (73%), lymphopenia (6%)), and platelets (thrombocytopenia, 64%) which are commonly observed in patients receiving oxaliplatin alone or in combination with fluorouracil and leucovorin. The National Comprehensive Cancer Network specifically states that platinum-based chemotherapies such as oxaliplatin induce these adverse effects primarily by directly impairing hematopoiesis in the bone marrow, and this effect can accumulate with repeated cycles of therapy. The goal of this study was to connect the changes in the transcriptome with the hematological toxicity.

Despite widespread acknowledgment of oxaliplatin-induced cytopenia, the precise molecular and transcriptional programs underlying lineage-specific hematopoietic disruption remain poorly defined. Most prior studies have examined these effects either through peripheral blood counts or histopathology alone. However, emerging transcriptomic technologies offer a deeper, systems-level understanding of how oxaliplatin alters gene expression within bone marrow lineages to impair blood cell development. In this study, we combine conventional hematological measurements with bulk RNA sequencing of murine bone marrow to provide an integrated view of oxaliplatin’s impact across all major hematopoietic lineages.

Combining quantitative blood cell measurements with lineage-specific transcriptional signatures, we demonstrate that oxaliplatin induces a coordinated suppression of hematopoiesis that is strongest in the erythroid and lymphoid lineages, followed by monocytes, with megakaryocyte pathways showing compensatory transcriptional activity despite decreased platelet counts. Interestingly, neutrophil output appears relatively spared, both transcriptionally and phenotypically. The combined data enable a mechanistic dissection of oxaliplatin’s hematologic toxicity and points to potential pathways of partial recovery and resistance.

## Methods

### Animal care and experimental protocol

All experiments were carried out under Washington U. IACUC-approved protocol. Male and female 10-week-old C57BL/6 mice (Charles River Laboratory) were housed at room temperature and maintained on a 12:12 hour light/dark cycle with free access to water and food. Mice were randomly assigned to two groups: injection of 10 mg/kg oxaliplatin in 5% dextrose (Sandoz) or an equal quantity of vehicle control (5% dextrose) as we have previously reported (4). Drugs were delivered intraperitoneally (i.p.) for 8 weeks. Blood samples from cheek bleeds were collected at the start of the experiment, 4 weeks, and 8 weeks. Mouse body weights were monitored throughout the experiments.

The 10 mg/kg oxaliplatin dose used in this study was determined according to the recommended regimen used in clinical practice as previously described (4). Oxaliplatin dosing for humans (85 mg/m^2^ biweekly) is based on a mg/surface area (m^2^) basis to account for differences in patient heights and masses (5). The mouse equivalent dose (mg/kg) was similarly calculated based on body surface area. The quantity of drug delivered to mice was adjusted to each mouse’s individual body mass at the time of injection to ensure the dose did not exceed clinically relevant concentrations.

### Blood analysis

Hematological parameters were determined on mouse blood at weeks 0, 4 and 8 (day of sacrifice) of the experiment. Blood was collected from the submandibular vein via lancet puncture. Mice were restrained by hand at the nape of the neck. Up to 100 μL was collected and stored in EDTA-treated tubes on ice for less than an hour to avoid coagulation. Blood samples were tested with a Hemavet 1700 System (Drew Scientific) for complete blood counts (CBC), leukocyte differential counts, and red blood cell (RBC) parameters.

Estimates of individual leukocyte numbers were calculated from the WBC counts from the CBC and the differential count percentages. The following formula was used to determine the numbers of leukocyte types:

Eq. 1 $$N_{\text{type}}=\text{WBC count}\times \text{Diff}{\%}_{\text{type}}/100$$

Where *N* is the number of a particular leukocyte type, *WBC count* is the number of white blood cells from the CBC, *Diff%*_*type*_ is the percentage of a particular type of leukocyte.

### Cytokine measurements

Blood samples collected in EDTA-treated tubes were stored on ice until centrifugation (10,000 rpm, 4°C, 10 min). The supernatant was stored at −80°C. The cytokine levels were assessed using a commercially available, customized magnetic bead multiplex kit (Procartaplex Mouse Simplex beads, ThermoFisher). The assays were performed according to the manufacturer recommended protocol, with the modification of performing an overnight 4°C incubation for the multiplex cytokines. Milliplex Analyst 5.1.0.0 (Merck Millipore) was used to analyze and calculate the cytokine concentration from the median fluorescent intensity of each bead.

### Bone Marrow Extraction

Six days after the last oxaliplatin treatment, mice were euthanized via isoflurane induction and cervical dislocation. Bone marrow was harvested from tibia and femur by placing the cut end down in a perforated 0.5 mL centrifuge tube inserted in a 1.5 mL tube pre-filled with 500 mL RNA later solution, followed by centrifuge at 5,000g for 30s. To extract RNA, 1mL TRIzol (Invitrogen 15596026) reagent was used per 100 mL RNA later-bone marrow mixture. RNA was further purified on Pure link RNA Mini Kit columns (Invitrogen 12183018A).

### RNA sequencing and data analysis

RNA sequencing (RNA-seq) followed our established protocol (4). The RNA quality was assessed using the Agilent TapeStation 4200 System. Samples with an RNA integrity number (RIN) greater than 7 were used for sequencing. A cDNA library was prepared following the manufacturer’s protocol using the Clontech SMARTer method. The library was indexed, pooled, and sequenced on an Illumina NovaSeq 6000.

RNA-seq reads were aligned via the Ensembl release 101 (European Bioinformatics Institute) using STAR version 2.5.1a (6). Aligned reads were imported into Partek Flow (Partek Inc.) for downstream analysis. Briefly, gene counts were derived from the number of uniquely aligned unambiguous reads and normalized using the median ratio method. DEGs were identified with the DESeq2 package implemented in Partek Flow. DEGs with an FDR < 0.05 and absolute value fold change (|FC|) > 1.5, total 3691 DEGs), were utilized for KEGG analysis (7), g: Profilers using a custom code, STRING-db (8), Gene Ontology (9).

### Bioinformatics

thematicGO: Standard Gene Ontology (GO) enrichment methods often yield extensive lists of individual terms, complicating identification of coherent biological processes across complex datasets. To address this limitation, we applied a thematic grouping strategy (10) that consolidates enriched GO terms into biologically meaningful themes using a curated, keyword-based assignment approach. For example, the *Cell-cycle & Apoptosis* theme includes terms containing keywords such as *cell cycle*, *mitotic*, *chromosome*, *checkpoint*, *DNA replication*, *nuclear division*, *apoptosis*, *programmed cell death*, and *caspase*. A complete list of themes and associated keywords is provided in Supplemental Table S2. thematicGO enrichment analysis was performed on DEGs (FDR < 0.05, |FC| > 1.5; n = 3,691) using the *Mus musculus* genome as the background.

### Correlation analysis

Correlation analysis was performed using Inter Variability Cross-Correlation Analysis (IVCCA) software package that we have previously developed (11).

## Results

### Oxaliplatin affects a large number of genes in bone marrow: overall effect

**Figure 1.**
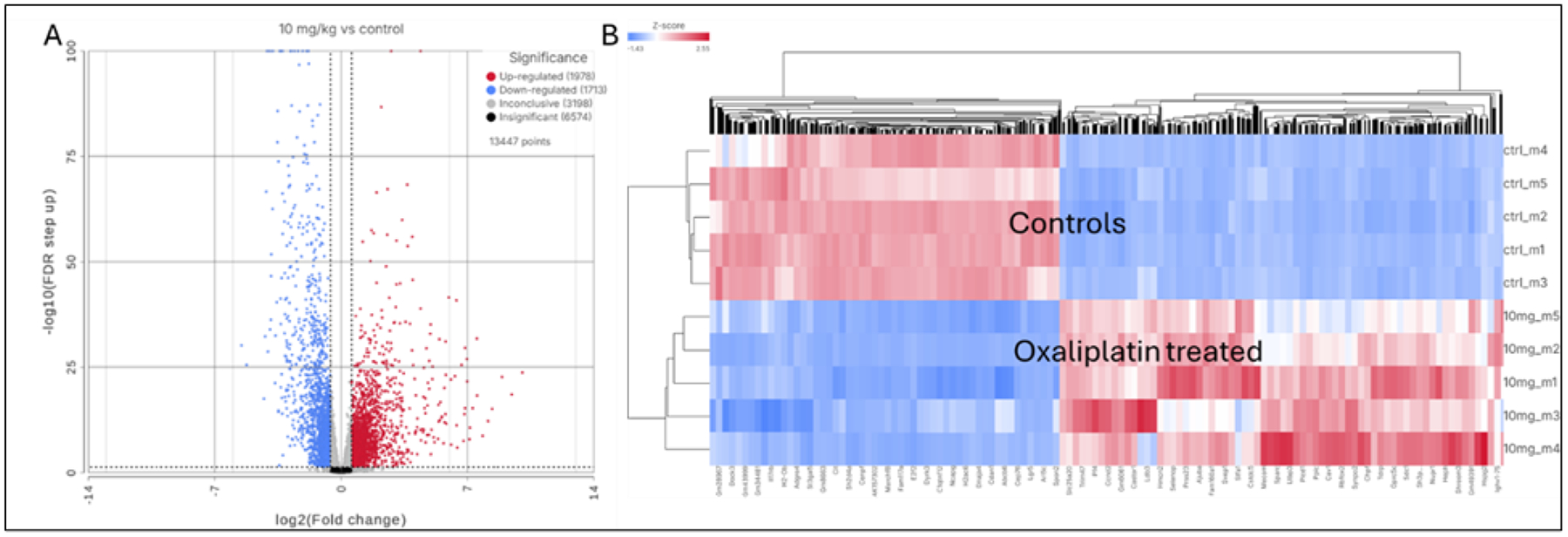
Transcriptional profiling of bone marrow in mice treated with 10 mg/kg oxaliplatin vs controls, weekly for 8 weeks. A. Volcano plot of gene expression affected by oxaliplatin treatment. (·) Blue dots: downregulated by oxaliplatin with FC < −1.5 and FDR<0.05. (·) Red dots: Upregulated by oxaliplatin with FC > 1.5 and FDR <0.05. Dashed vertical lines represent the −1.5x and 1.5x FC borders. Dashed horizontal line represent the FDR = 0.05. B. Heatmap of DEGs with |FC| > 1.5 affected by oxaliplatin treatment. First 5 rows correspond to the control untreated group, lower rows correspond to mice threated with 10 mg/kg oxaliplatin weekly for 8 weeks.

Bulk RNA-seq analysis of bone marrow identified more than 13,400 genes in control and oxaliplatintreated mice. For analysis, the RNA-seq results were separated based on the FDR score < 0.05 and a |FC| > 1.5 leading to 3,691 DEGs (Supplemental Table S1 and Figure 1A). Heatmap analysis of these DEGs demonstrated clear and distinct clustering of oxaliplatin-treated vs control groups (Figure 1B), indicating a consistent effect of the treatment on gene expression among individual mice.

**Figure 2.**
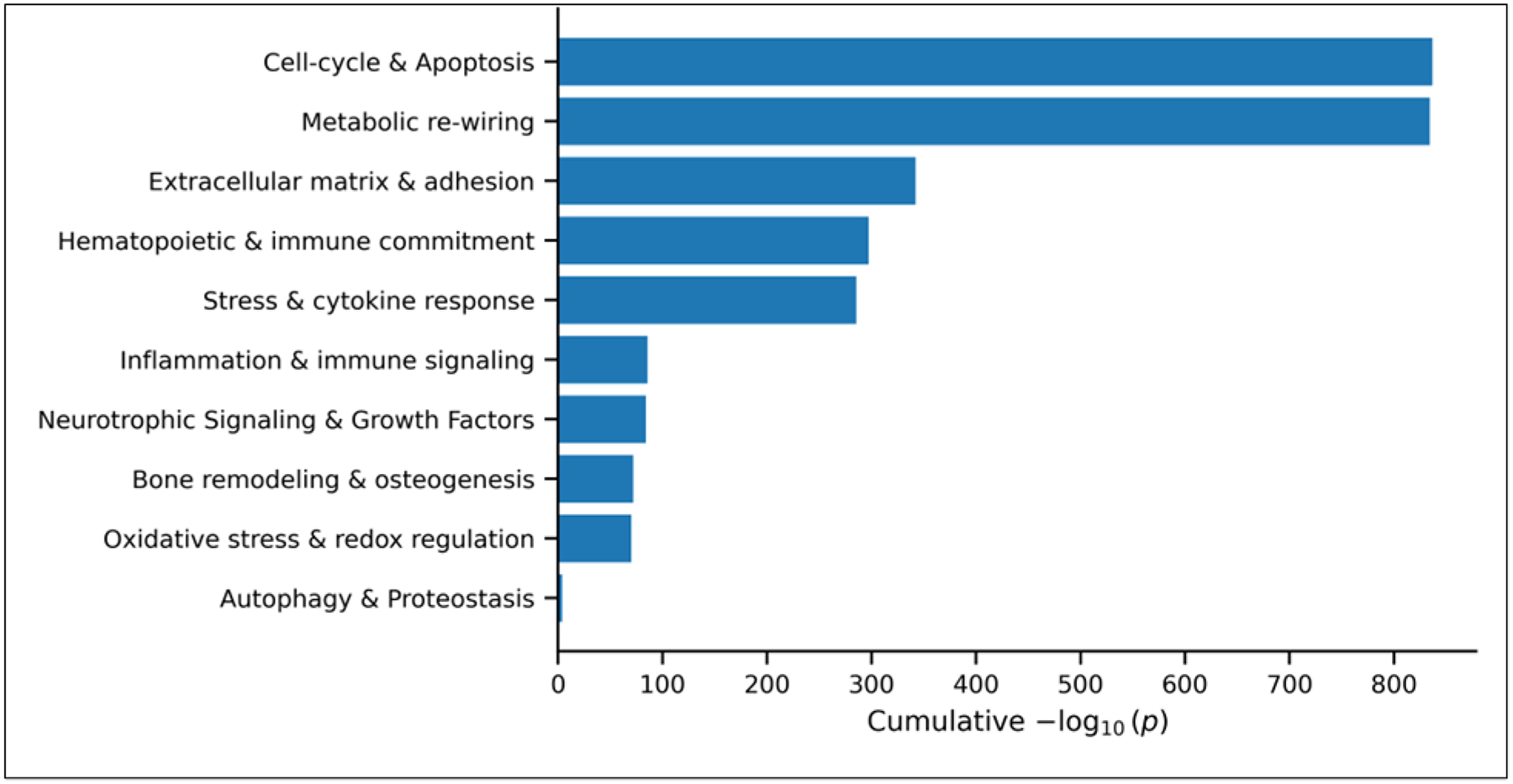
thematicGO enrichment of DEGs in bone marrow after oxaliplatin treatment based on 3,691 DEGs (FDR<0.05, |Fold change|>1.5). Bar plot shows the cumulative enrichment scores (−log_10_ of p-values) for grouped biological themes derived from the significantly enriched Gene Ontology (GO) terms with p-value less than 0.01. GO terms were classified into predefined categories based on keyword matching. The names of the terms for each theme are provided in the Supplemental Section, Table S2.

The enrichment analysis in g:Profiler showed an extremely strong signal with statistical significance p ≈ 10^−36^ – 10^−32^ for broad developmental and morphogenetic programs. Drilling into the significant rows (p < 10^−4^) revealed five tightly-connected themes: i) Cell-cycle regulation & apoptosis; ii) Metabolic re-wiring, iii) Extracellular matrix remodeling & cell-adhesion, iv) Hematopoietic & immune lineage commitment and v) Response to stress & cytokines, ( Figure 2).

### Oxaliplatin and cell cycle arrest, apoptosis, and metabolic rewiring

Transcriptomic analysis of bone marrow from oxaliplatin-treated mice revealed pronounced alterations in genes regulating cell cycle progression, apoptosis, and cellular metabolism (Figure 2). Among the differentially expressed genes, *Cdkn1a* (encoding p21) was one of the most strongly upregulated (FC = +21.1), consistent with activation of DNA damage response pathways and G1/S cell cycle arrest. This was accompanied by downregulation of the S-phase–associated transcription factor *E2f2* (FC = −3.4) (12) and altered expression of Cyclin D family members (*Ccnd2* and *Ccnd1*), indicating broad disruption of cell-cycle control (13). Integrative correlation analysis (IVCCA) identified *Cdkn1a* as one of the most highly connected genes in the dataset, placing it among the top 2.5% of correlated DEGs.

Oxaliplatin treatment also induced transcriptional changes consistent with metabolic rewiring, including upregulation of multiple glycolytic enzymes (e.g., *Pfkl*, *Tpi1*, *Ldha*; Figure S2), suggesting a shift toward glycolytic metabolism. Expression of *Cdkn1a* was positively associated with these metabolic changes and negatively associated with genes involved in calcium handling and lipid remodeling, consistent with coordinated suppression of biosynthetic and homeostatic pathways.

Notably, *Sirt1* expression was reduced in bone marrow following oxaliplatin treatment (FC = −1.36, FDR = 1.11E-06), consistent with reported NAD^+^ depletion and suppression of SIRT1 activity in oxaliplatin-treated cells (14). Given SIRT1’s role in the development of hematopoietic stem cells (15) as well as restraining p53 signaling (16) and supporting lymphoid development under stress, its downregulation may contribute to the *Cdkn1a*-centered cell-cycle arrest and immune suppression observed here.

In parallel, several canonical pro-apoptotic genes were significantly induced, including the death receptor *Tnfrsf10b* (17) and p53-responsive mediators of intrinsic apoptosis (*Pmaip1*, *Bax*, *Bbc3*), indicating activation of both extrinsic and intrinsic apoptotic programs (18, 19). *Cdkn1a* expression was strongly associated with these pro-apoptotic signatures, whereas the anti-apoptotic regulator *Sesn1* was downregulated and inversely correlated with *Cdkn1a*.

Overall, these findings indicate that *Cdkn1a* serves as one of the hub genes coordinating several critical pathways in bone marrow in response to oxaliplatin including cell cycle arrest, apoptosis, and metabolic reprogramming in response to chemotherapy-induced stress.

### Oxaliplatin treatment does not cause a major shift in hematopoietic differentiation

Although oxaliplatin affects a larger number of genes in the bone marrow this treatment did not appear to cause a major shift in the transcription factors associated with hematopoietic differentiation. Among key lineage-defining transcription factors, only *Ikzf1*, a critical regulator of blood cells commitment (20) was found to be moderately downregulated (FC = −1.51) while other hematopoietic lineage decisions genes, including *Spi1* (PU.1), *Gata2*, *C/Ebpα*, and *Notch1*, remained unchanged, potentially due to recovery. This suggests that while oxaliplatin may affect lymphoid potential, particularly B- and T-cell progenitors (see below), it does not broadly disrupt the equilibrium between myeloid and lymphoid differentiation programs.

### Oxaliplatin induces severe anemia in mice and causes a major change in late stages of erythroid maturation

**Figure 3.**
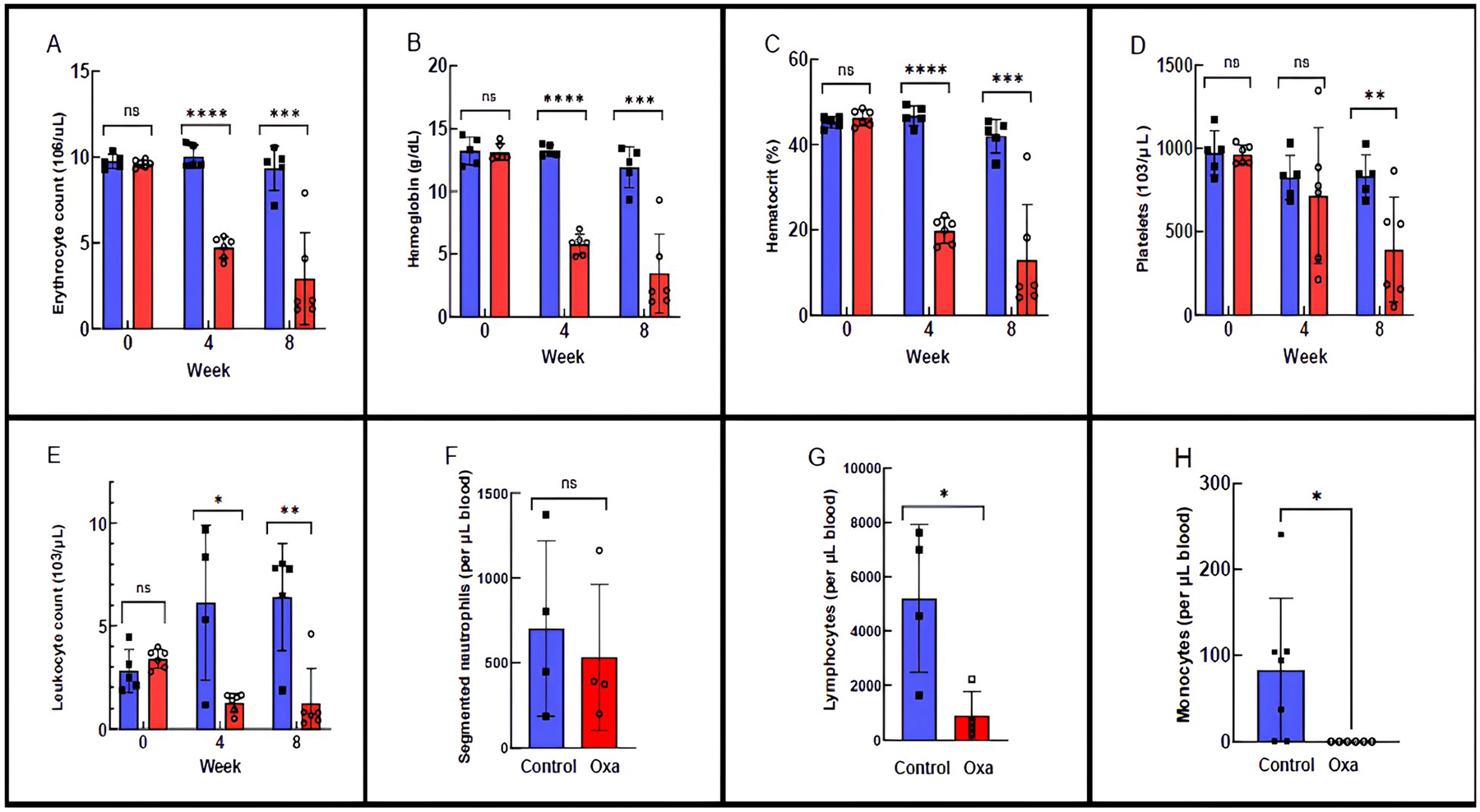
Effects of oxaliplatin on mouse blood parameters. A). Erythrocyte count. B). Hemoglobin. C). Hematocrit. D). Platelets. E). Leukocyte counts. F). Calculated segmented neutrophil counts. G). Calculated lymphocyte counts. H). Calculated monocyte counts. Bars represent the average ± STD with individual data points. T-test results are indicated by brackets. Statistical significance marks: *=p<0.05, **=p<0.01, *** = p<0.001, **** = p<0.0001

Oxaliplatin treatment resulted in profound, dose-dependent anemia, characterized by marked reductions in erythrocyte counts, hemoglobin concentration, and hematocrit (Figure 3A–C). Erythrocyte counts declined from the normal murine range (7,800–10,500 k/μL) to below 3,000 k/μL, while hemoglobin levels fell to as low as 3.45 g/dL in some animals, corresponding to severe (Grade 3–4) anemia (21). Four of six oxaliplatin-treated mice exhibited hemoglobin concentrations below clinically concerning thresholds (22, 23) at which erythropoiesis-stimulating agents or transfusion are typically recommended. Platelet counts were also reduced to approximately 390 k/μL, consistent with moderate thrombocytopenia.

These hematologic abnormalities were supported by transcriptomic evidence of impaired late-stage erythroid maturation in the bone marrow. Key hemoglobin and erythroid structural genes (*Hba-a1*, *Hbb-bs*, *Alas2*, *Spta1*, *Ank1*, *Slc4a1*) (24–27) were strongly downregulated, indicating disruption of red blood cell membrane integrity and heme biosynthesis. Notably, expression of the erythropoietin receptor (*Epor*) was reduced (FC = −2.27) despite a ~3000-fold increase in renal *Epo* expression (GSE301439), indicating erythropoietin resistance at the level of erythroid progenitors.

Although oxaliplatin significantly reduced circulating erythrocyte numbers, red blood cell morphology was largely preserved. Transient decreases in mean corpuscular volume and hemoglobin were observed at week 4 but returned to baseline by week 8 (Figure S1), indicating that anemia was driven primarily by reduced erythrocyte production rather than defective erythrocyte maturation. Consistent with coordinated erythroid suppression, expression of erythroid maturation genes strongly correlated with hemoglobin gene expression (Supplementary Table S1).

### Circulating platelets are reduced despite transcriptional activation of thrombopoiesis

Platelet counts in oxaliplatin-treated mice remained stable at week 4 but declined by approximately 50% by week 8, reaching 383 k/μL corresponding to ~44% of the lower limit of normal and consistent with Grade 2 thrombocytopenia (Figure 3D). In contrast, bone marrow RNA-seq revealed upregulation of multiple megakaryocyte and platelet-associated genes, including *Pf4*, *Gp1ba*, *Ppbp*, *Mpl*, and *Itga2b*, indicating transcriptional activation of thrombopoietic programs despite reduced circulating platelet numbers.

Expression of *Thpo*, the primary cytokine regulating megakaryocyte differentiation, was modestly reduced in the kidney (FC = −1.41), suggesting altered systemic regulation of platelet production under oxaliplatin treatment.

## Lymphoid Lineages and Immune Suppression

The number of leukocytes in the mice was heavily impacted by oxaliplatin (Figure 3E) from the normal leukocyte count average of 6,390/mL within the range 2,000–10,000/mL (28) to level of 1,273/mL.

### Cytokines revealed a pro-inflammatory shift

**Figure 4.**
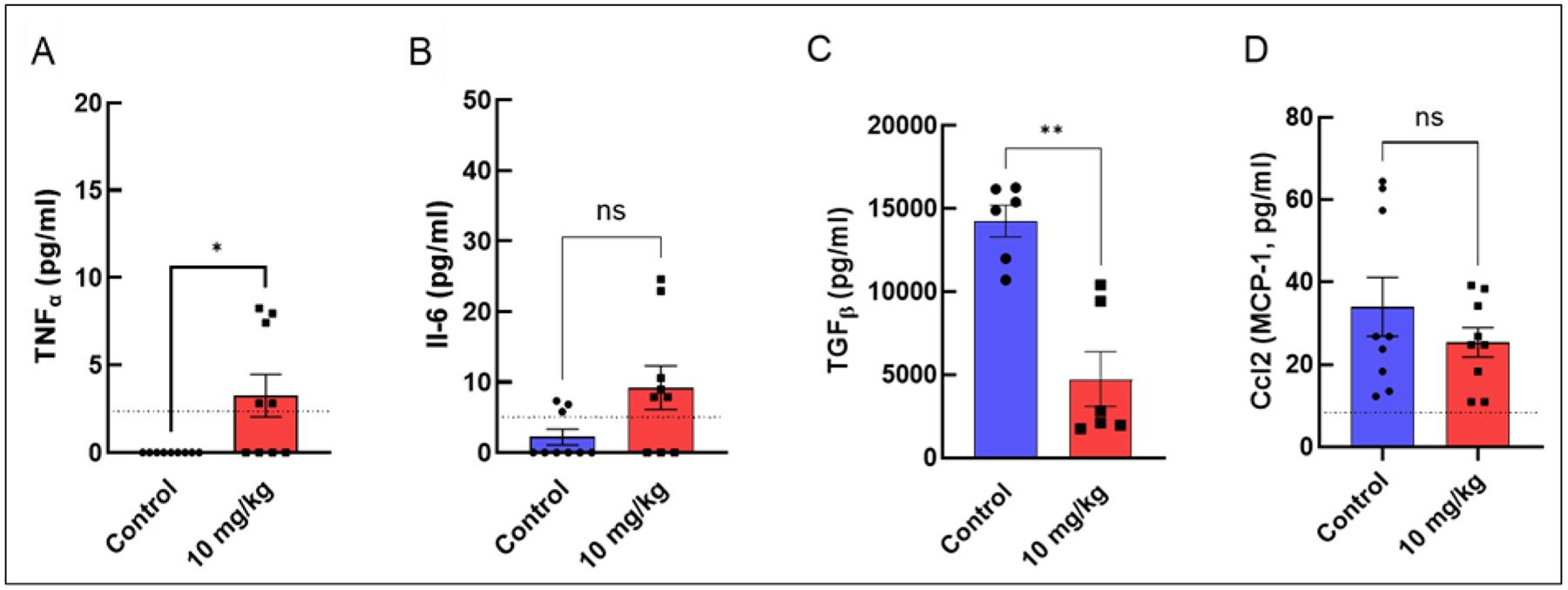
Oxaliplatin affects the level of pro-inflammatory cytokines A) TNFα and B) IL-6 as well as anti-inflammatory cytokines C) TGF-β and D) CCL2 (MCP-1). Means ± SEM. Unpaired T-tests. Dashed lines represent detection limits for each cytokine with the exception of TGF-β (0.94 pg/mL). * p<0.05, ** p<0.01

Cytokine profiling of plasma revealed a pro-inflammatory shift in oxaliplatin-treated mice, characterized by significant upregulation of TNF-α and downregulation of the anti-inflammatory cytokine TGF-β (Figure 4). IL-6 did not reach statistical significance but showed a consistent upward trend. TNF-α is known to impair erythropoiesis (29) by inhibiting EPO-mediated signaling, while IL-6 has been implicated in suppression of erythroid maturation through downregulation of *Slc4a1*(30).

TGF-β plays a central role in maintaining HSC quiescence and regulating hematopoietic homeostasis (31). Notably, while circulating TGF-β levels were reduced, expression of all three *Tgfb* isoforms (*Tgfb1/2/3*) was increased in bone marrow, suggesting compartment-specific regulation of inflammatory signaling.

Consistent with this inflammatory environment, peripheral blood analysis revealed a broad reduction in leukocytes, including segmented neutrophils and lymphocytes. Transcriptomic analysis further supported pan-lineage immune suppression, with downregulation of the myeloid differentiation factor *Irf7* and the B-cell development marker *Cd19*, indicating impaired differentiation across both myeloid and lymphoid compartments.

### Innate Immune Cells

Analysis of innate immune populations revealed lineage-specific effects of oxaliplatin. Segmented neutrophil counts were not significantly altered by week 8 (Figure 3F), consistent with transcriptomic data showing preserved expression of key neutrophil differentiation and function genes, including *Cebpa*, *Gfi1*, *Mpo*, and *Elane* (32–34). These findings indicate that neutrophil development from the myeloid lineage is largely preserved at the transcriptional level following oxaliplatin treatment.

In contrast, monocyte counts were significantly reduced (Figure 3H), accompanied by downregulation of *Irf8*, a transcription factor essential for monocyte, macrophage, and dendritic cell differentiation (35). This suggests selective vulnerability of the monocyte lineage within the innate immune compartment.

Eosinophils and basophils remained low or undetectable in both control and oxaliplatin-treated mice, indicating minimal impact of oxaliplatin on these minor granulocyte populations.

### Adaptive Immune Cells

Lymphocyte counts declined sharply from approximately 7,000/μL in control mice to ~1,200/μL following oxaliplatin treatment (Figure 3G), indicating severe lymphopenia. This phenotype was mirrored by transcriptomic analysis, which revealed broad suppression of lymphoid lineage genes, with the most pronounced effects observed in B cells and CD4^+^ T cells. Key B-cell markers (*Cd79a*, *Cd79b*, *Pax5*, *Ms4a1*) and immunoglobulin genes (*Ighm*, *Ighd*) were strongly downregulated, consistent with impaired B-cell development. Markers of CD4^+^ T cells (*Cd4*) were also reduced, whereas more general T-cell markers such as *Cd3e* were relatively preserved, suggesting lineage- and maturation-dependent sensitivity to oxaliplatin.

Expression of *Il7r*, a receptor critical for lymphoid development, was markedly reduced despite significant upregulation of *Il7*, potentially reflecting a compensatory response to lymphoid depletion. In contrast to B and CD4^+^ T cells, NK cell–associated genes were only modestly affected, indicating relative preservation of this lineage (Table S1). Correlation analysis (Table S2) demonstrated coordinated downregulation of T-, B-, and NK-associated markers, consistent with a synchronized suppression of lymphoid maturation within the bone marrow.

Together, these findings indicate that oxaliplatin induces a profound transcriptional collapse of lymphoid differentiation, resulting in reduced lymphocyte production and potential impairment of adaptive immune function and therefore increased infection risk in the patient.

### Signs of bone marrow recovery

Hematopoietic suppression induced by oxaliplatin is often transient in clinical settings, with recovery of blood counts observed after dose adjustment or treatment cessation (36). Consistent with this pattern, we observed evidence of partial recovery of specific hematopoietic lineages in oxaliplatin-treated mice (Table S1).

Megakaryocyte-associated markers such as *Itga2b* (CD41) were modestly upregulated (FC = +1.53), suggesting rebound activation of the platelet lineage. Markers associated with granulocytic and monocytic populations, including *Fcgr2b* (FC = +3.01) and *Ly6c1* (+2.29), were also increased, indicating recovery within the myeloid compartment. In addition, hematologic analysis revealed anisocytosis in oxaliplatin-treated mice, consistent with the presence of immature erythroid cells and ongoing bone marrow regeneration (28).

## Discussion

### Challenges in defining transcriptomic mechanisms of oxaliplatin-induced bone marrow toxicity

Although oxaliplatin-associated anemia and immunosuppression are well documented in both animal models (37) and patients (38), the transcriptomic basis underlying these hematologic effects has remained poorly characterized. One contributing factor has been the technical challenge of generating high-quality RNA-seq data from bone marrow, a tissue prone to rapid RNA degradation due to high endogenous RNase activity and complex cellular composition. Using optimized stabilization and processing protocols, we obtained high-quality bone marrow RNA with consistently high integrity, enabling robust transcriptomic analysis and direct integration of molecular changes with hematologic phenotypes

### Oxaliplatin induces a multi-hit disruption of the bone marrow niche

Our transcriptomic analysis reveals that oxaliplatin exerts hematopoietic toxicity through a coordinated, multi-hit mechanism that disrupts the bone marrow niche at multiple levels. Central to this response is marked upregulation of *Cdkn1a* driven by p53 activation, coupled with downregulation of *E2f2*, consistent with G1/S cell-cycle checkpoint arrest. This blockade would be expected to impair proliferation across multiple hematopoietic lineages. Concomitant suppression of hemoglobin genes (*Hba*, *Hbb*) and erythroid maturation programs further supports a failure of red blood cell production rather than increased peripheral destruction.

Remarkably, these transcriptional defects persist despite a striking ~3000-fold upregulation of renal *Epo* expression, indicating a state of erythropoietin resistance. Reduced expression of the erythropoietin receptor (*Epor*) and loss of erythroid progenitors in the bone marrow likely underlie this uncoupling between systemic EPO signaling and erythropoiesis. This phenotype resembles features of bone marrow failure syndromes such as myelodysplastic syndromes, in which elevated circulating EPO fails to restore effective erythropoiesis due to impaired receptor signaling or progenitor dysfunction (39). Such EPO resistance may help explain the persistence and severity of anemia observed clinically in patients receiving oxaliplatin (40).

### Selective suppression of adaptive immunity and inflammatory imbalance

In addition to erythropoiesis, oxaliplatin profoundly impairs lymphoid development, as evidenced by downregulation of B-cell markers (*Cd79a*, *Cd79b*, *Pax5*, *Ms4a1*) and T-cell–associated genes (*Cd4*, *Il7r*). Importantly, early hematopoietic transcription factors remained largely unchanged, suggesting that oxaliplatin primarily disrupts downstream differentiation and maturation rather than lineage commitment. This selective vulnerability of adaptive immune lineages likely contributes to prolonged immunosuppression.

Cytokine profiling further revealed a pro-inflammatory shift characterized by increased circulating TNF-α and reduced TGF-β. Such an imbalance may exacerbate hematopoietic dysfunction by promoting inflammatory stress while removing key regulatory signals that normally preserve stem cell quiescence and homeostasis. Together, these findings suggest that inflammatory signaling cooperates with intrinsic transcriptional defects to destabilize the bone marrow environment.

### Hierarchy of lineage vulnerability

**Figure 5.**
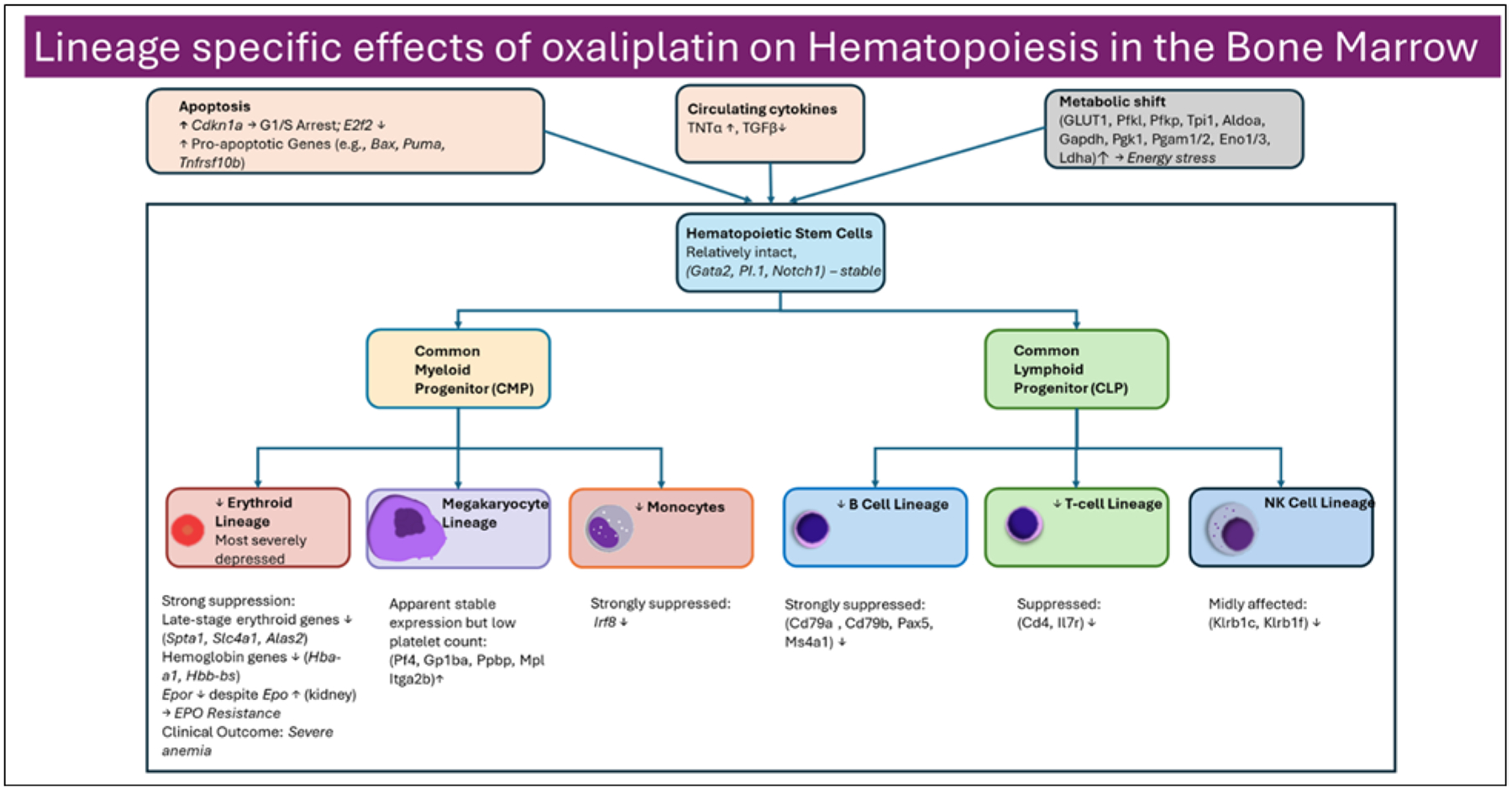
Lineage specific effects of oxaliplatin on hematopoiesis in the bone marrow. Oxaliplatin disrupts both erythropoiesis and lymphopoiesis, through apoptosis and metabolic shift primarily affecting the late stages of red blood cell development and the differentiation of lymphoid progenitors, while early multipotent hematopoietic cells remain relatively intact.

Integration of hematologic, transcriptomic, and cytokine data reveals a clear hierarchy of sensitivity to oxaliplatin-induced toxicity. Erythroid cells are most severely affected, followed by lymphoid populations, whereas granulocytic lineages such as neutrophils remain relatively preserved. This pattern is consistent with differential proliferative demands and lineage-specific susceptibility to cell-cycle arrest and metabolic stress. Overall, these data support a model in which oxaliplatin disrupts the bone marrow niche through combined effects on cell-cycle control, differentiation, metabolism, and inflammatory signaling (summarized in Figure 5).

### Comparison to Human Data: Concordance and Divergence

Our findings in the murine model broadly recapitulate the hematologic toxicities observed in oxaliplatin-treated patients, supporting the translational relevance of this study. Clinically, oxaliplatin is associated with high rates of anemia, leukopenia, and thrombocytopenia, with variable effects on lymphocyte populations depending on treatment regimen and patient characteristics (3, 5, 41–46). Consistent with these observations, oxaliplatin-treated mice exhibited profound anemia, leukopenia, thrombocytopenia, and monocytopenia, accompanied by marked transcriptional suppression of erythroid and lymphoid differentiation programs in the bone marrow.

One area of divergence is the relative preservation of neutrophils in mice. While human patients frequently experience neutropenia, especially in the context of combination regimens like FOLFOX, segmented neutrophil counts and key transcriptional regulators of granulopoiesis (*C/Ebpα, Gfi1, Elane*) remained stable in our mouse model. This discrepancy reflects species-specific differences in myeloid lineage sensitivity, as well as timing of sampling, or compensatory responses not captured in the current protocol.

Another difference is the magnitude of lymphoid suppression. While lymphopenia is reported in a minority of oxaliplatin-treated patients, our mouse model revealed a robust suppression of both B and T lymphocyte development at the transcriptional level. This may be due to differences in cumulative dose, absence of co-administered drugs (e.g., 5-FU or leucovorin (42)), or inherent differences in immune cell turnover rates between mice and humans.

Importantly, our identification of erythropoietin resistance characterized by marked upregulation of renal *Epo* expression coupled with reduced bone marrow *Epor* parallels clinical observations in which erythropoiesis-stimulating agents show variable or limited efficacy in correcting chemotherapy-induced anemia. EPO insensitivity is a recognized feature of several bone marrow failure states (47, 48), and response rates to ESA therapy in patients receiving platinum-based chemotherapy remain inconsistent (22, 49). Our data provide molecular evidence supporting this clinical phenomenon.

In summary, the mouse model faithfully captures several key hematologic toxicities of oxaliplatin observed in patients, particularly anemia and platelet suppression, while also uncovering potential mechanisms such as EPO resistance and lymphoid collapse that may be underappreciated or variable in clinical settings.

## Conclusion

Our study demonstrates that oxaliplatin induces a lineage-specific suppression of hematopoiesis driven by coordinated cell cycle arrest, metabolic dysregulation, and disrupted cytokine signaling. Among all lineages, erythropoiesis and lymphopoiesis were the most severely affected, as evidenced by marked reductions in peripheral cell counts and transcriptional repression in bone marrow. Despite a massive (~3000-fold) compensatory increase in renal erythropoietin (*Epo*) expression, bone marrow *Epor* was downregulated, indicating a state of EPO resistance that may underlie the persistent anemia seen in treated mice.

Additionally, the suppression of B- and T-cell lineage genes and downregulation of *Il7r* suggest a significant disruption of adaptive immunity, potentially compromising host defense. Myeloid cells showed a dichotomous response: while neutrophils were preserved, monocytes were markedly suppressed, and megakaryocyte-associated transcripts were upregulated despite declining platelet counts - suggesting an incomplete or ineffective compensatory response, or an autoimmune complication as seen in human patients.

Together, these findings refine our understanding of oxaliplatin toxicity as a multi-hit disruption of the bone marrow niche and immune environment. Future studies using single-cell resolution and functional assays will be critical for validating these mechanisms and guiding interventions to restore hematopoietic balance and mitigate off-target toxicities.

## Supplementary Material

Supplemental Materials

Supplemental Table S1

Supplementary Material

Supplementary Table 1

## Data Availability

RNA sequencing data on bone marrow and kidney has been deposited to GEO under session number GSE301439: https://www.ncbi.nlm.nih.gov/geo/query/acc.cgi?acc=GSE301439 thematicGO code in Python is available from Github: https://github.com/MikhailBerezin/thematicGO
