# Supplementary material for: Lineage-Specific Disruption of Hematopoiesis by Oxaliplatin: Mechanisms of Erythropoietin Resistance and Immune Suppression
Source: J Hematol Oncol Res. Author manuscript; Available in PMC 2026 Mar 22. (PMC13005433; doi:10.14302/issn.2372-6601.jhor-25-5944)
Supplement: Supplemental Materials [file NIHMS2154647-supplement-Supplemental_Materials.docx]

**Supplemental INFORMATION**

**Title: Lineage-Specific Disruption of Hematopoiesis by Oxaliplatin: Mechanisms of Erythropoietin Resistance and Immune Suppression**

Leland C. Sudlow^a*^, Junwei Du^a,b*^, Kiana Shahverdi^a^, Haiying Zhou^a^, Mikhail Y. Berezin^a,b^

**Institutional Affiliations**: ^a^Mallinckrodt Institute of Radiology, Washington University School of Medicine St. Louis, MO 63110, USA; ^b^Institute of Materials Science & Engineering Washington University, St. Louis, MO 63130, USA

**FIGURES**

**
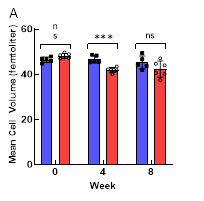

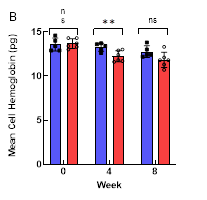

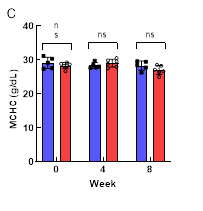
**

|  |  |  |
| --- | --- | --- |

**Figure S1** **Effects of oxaliplatin on mouse erythrocyte parameters.** **A**). Erythrocyte mean cell volume (fL). **B**). Erythrocyte mean cell hemoglobin (pg). **C**) Erythrocyte mean cell hemoglobin concentration (g/dL). Bars represent the average ± STD with individual data points. T-test results are indicated by brackets. Statistical significance marks: *=p<0.05, **=p<0.01, *** = p<0.001, **** = p<0.0001


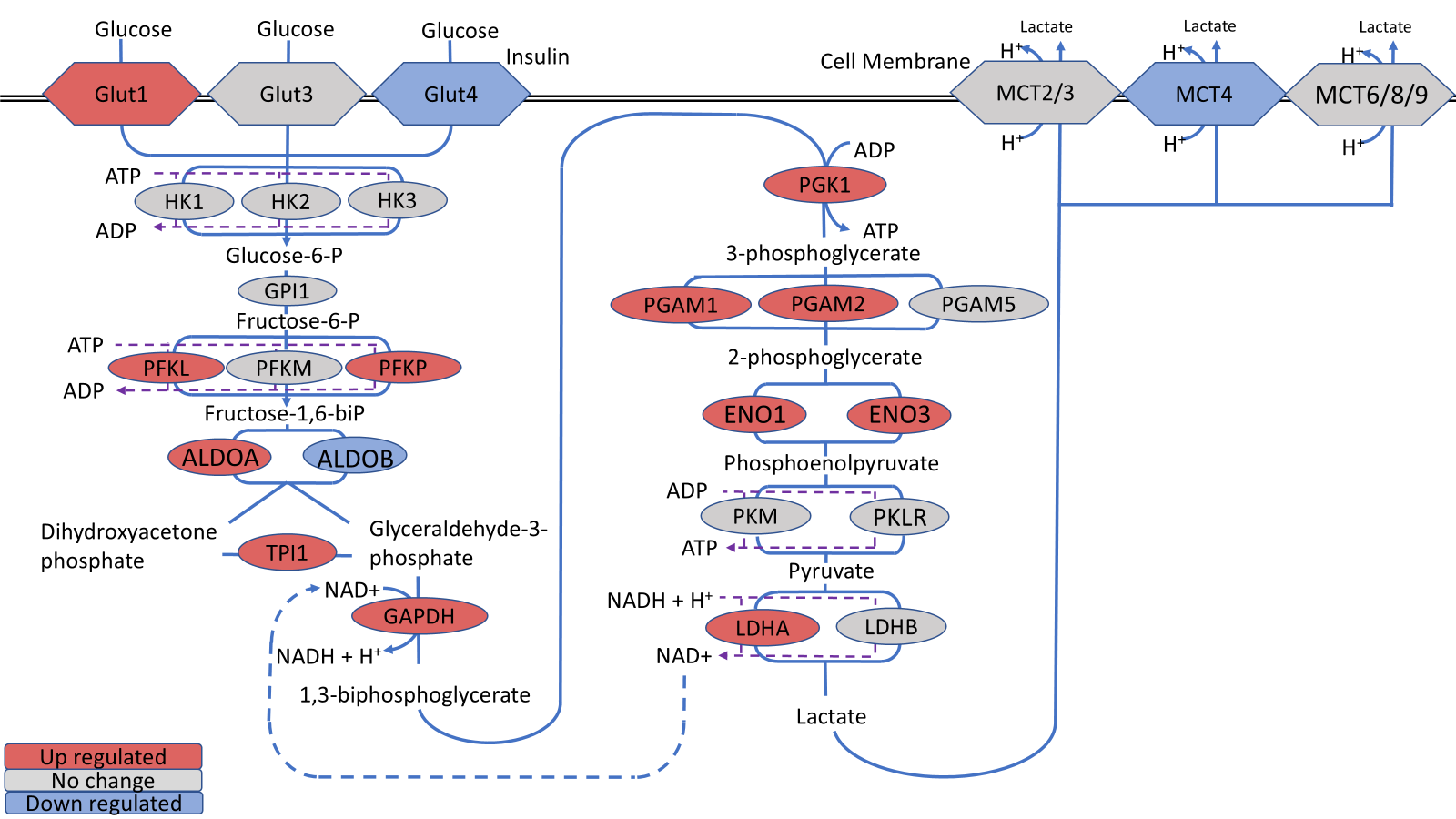


**Figure S2: Glycolysis in bone marrow is upregulated in the oxaliplatin treated mice.** Abbreviations of enzymes and proteins with encoded genes is in parenthesis: ALDOA, aldolase A (*Aldoa*); ALDOB, aldolase B (*Aldob*); ENO1/3, enolase 1and 3 (*Eno1*and *Eno3*); GAPDH, glyceraldehyde-3-phosphate dehydrogenase (*Gapdh*); GLUT1, glucose transporter 1 (*Slc2a1*); GLUT3, glucose transporter 3 (*Slc2a3*); GLUT4, insulin-responsive glucose transporter 4; GPI1, glucose-6-phosphate isomerase 1 (*Gpi1*); HK1/2/3, hexokinase 1/2/3 (*Hk1, Hk2,* *Hk3*); LDHA, lactose dehydrogenase A (*Ldha*); LDHB, lactose dehydrogenase B *(Ldhb*); MCT1/3/6 monocarboxylic acid transporters 1/3/6 (*Slc16a1, Slc16a3, Slc16a6*); PGAM1/2/5, phosphoglycerate mutase 1/2/5 (*Pgam1, Pgam2, Pgam5*); PGK1, Phosphoglycerate Kinase 1 (*Pgk1*); PKFL, phosphofructokinase liver type (*Pfkl*); PKFM, phosphofructokinase muscle type (*Pfkm*); PKFP, phosphofructokinase platelet type (*Pfkp);* PKLR, pyruvate kinase, liver and RBC (*Pklr*); PKM, pyruvate kinase, muscle type (*Pkm*); TPI1, topoisomerase 1 (*Tpi1*). The schematics is in part based on KEGG Pathways. Red – upregulated genes, grey – unaffected genes, blue downregulated genes.

**TABLES**

**Table S1 Differentially expressed genes from bone marrow: oxaliplatin vs control.**

- (see a separate supplemental Excel file named **Table S1 Bone marrow 3691 DEGs FC 1.5**)

**Table S2 Keyword-Based Grouping of GO Terms into Biological Themes**

| **Theme** | **Keywords** |
| --- | --- |
| **Stress & Cytokine Response** | stress, interferon, cytokine, inflammatory, defense |
| **Inflammation & Immune Signaling** | inflammation, inflammatory, tnf, il-1, il-6, nf-kb, toll-like, interleukin, chemokine, ccl, cxcl, immune response, inflammasome, pattern recognition, pathogen response |
| **Oxidative Stress & Redox Regulation** | oxidative, redox, reactive oxygen, ros, nitrosative, nrf2, antioxidant, glutathione, superoxide, peroxidase, peroxiredoxin, sod, catalase, thioredoxin, oxidoreductase, hydrogen peroxide, peroxide, nitric oxide, peroxynitrite, nadph oxidase, mitochondrial ros, electron transport chain, mitochondrial dysfunction, oxidative damage, protein oxidation, lipid peroxidation, dna oxidation, redox imbalance |
| **Extracellular Matrix & Adhesion** | extracellular, matrix, adhesion, integrin, collagen, remodeling, fibronectin, laminin, basement membrane, mmp, matrix metalloproteinase, tenascin, focal adhesion, ecm, tissue remodeling, stromal, scaffold, matrisome, cell junction, cell adhesion, cell-matrix, desmosome |
| **Metabolic Re-wiring** | metabolic, oxidoreductase, catabolic, fatty, one-carbon, biosynthetic |
| **Hematopoietic & Immune Commitment** | hematopoiet, myeloid, lymphoid, leukocyte, granulocyte, erythro, megakary, erythropoiet, myelopoiet, thrombopoiet, lymphocyte, monocyte, neutrophil, eosinophil, basophil, platelet, erythrocyte, anemia, cytopenia, pancytopenia, thrombocytopenia, leukopenia, neutropenia, immune cell, blood cell, hematologic, hematopoiesis, stem cell, hsc |
| **Cell-Cycle & Apoptosis** | cell cycle, mitotic, chromosome, checkpoint, dna replication, nuclear division, apoptosis, programmed cell death, caspase |
| **Neurotrophic Signaling & Growth Factors** | neurotrophin, ngf, bdnf, ntf, trk, trka, trkb, gdnf, growth factor, igf, egf, fgf, receptor tyrosine kinase |
| **Immune–Neuronal Crosstalk** | microglia, macrophage, satellite glia, neuroimmune, neuroinflammation, cd11b, cd68, csf1, tslp, complement, ccr, cxcr |
| **Pain & Nociception** | pain, nociception, nociceptor, hyperalgesia, allodynia, trpv1, trpa1, scn9a, piezo, itch, sensory perception, neuropeptide |
| **Oxidative Phosphorylation & Mitochondria** | mitochondrial, oxidative phosphorylation, electron transport chain, atp synthase, complex I, respiratory chain, mitophagy |
| **Autophagy & Proteostasis** | autophagy, lysosome, proteasome, ubiquitin, protein folding, chaperone |
| **Bone Remodeling & Osteogenesis** | osteogenesis, bone formation, bone development, skeletal development, ossification, mineralization, bone mineralization, osteoblast, osteoblast differentiation, osteoblast proliferation, osteoid, bone matrix formation, alkaline phosphatase, runx2, osterix, sp7, collagen, osteoclast, osteoclast differentiation, osteoclastogenesis, bone resorption, trap, cathepsin k, bone remodeling, bone turnover, skeletal homeostasis, rank, rankl, opg, wnt signaling, beta-catenin, bmp, tgf beta, smad signaling, endosteal niche, bone marrow niche, osteolineage cell, hematopoietic niche, mesenchymal stem cell, stromal cell, bone matrix, hydroxyapatite, osteocalcin, osteopontin, sclerostin, osteoporosis, osteopenia, bone loss |
| **Neuronal Excitability & Synapse** *(disabled)* | axon, dendrite, synapse, neurotransmitter, vesicle, action potential, ion channel, potassium, sodium, calcium, glutamate, gaba, synaptic, neurogenesis, axonogenesis |
| **Myelination & Schwann Cell Biology** *(disabled)* | myelin, schwann cell, mbp, mpz, prx, pmp22, node of ranvier, myelination, myelin sheath, axon ensheathment, remyelination, demyelination, schwann cell differentiation, schwann cell proliferation, schwann cell migration, axon guidance, nerve regeneration |
| **Fibrosis** *(disabled)* | fibrosis, fibrotic, extracellular matrix, matrix organization, matrix remodeling, collagen, fibronectin, laminin, myofibroblast, tissue remodeling, tgf beta, smad signaling, emt, endmt |
| **Adipose Tissue Development** *(disabled)* | adipose tissue, adipogenesis, adipocyte, lipid storage, lipogenesis, ppar gamma, c/ebp, thermogenesis |
| **Cardiac & Muscle Function** *(disabled)* | heart, cardiac, cardiomyocyte, contraction, sarcomere, troponin, myosin, arrhythmia, heart failure, cardiomyopathy |

**Table S3 Correlation: Erythroid Maturation vs Hemoglobin Genes**

|  | **Hba-a1** | **Hba-a2** | **Hbb-bt** | **Hbb-bs** |
| --- | --- | --- | --- | --- |
| **Spta1** | 0.969 | 0.965 | 0.965 | 0.972 |
| **Slc4a1** | 0.985 | 0.982 | 0.982 | 0.987 |
| **Sptb** | 0.965 | 0.959 | 0.958 | 0.967 |
| **Ank1** | 0.948 | 0.942 | 0.943 | 0.950 |
| **Alas2** | 0.999 | 0.998 | 0.996 | 0.999 |

**Table S4 T cell (CD4) and B cell Marker Correlation**

|  | **T cells** | **B cells** | | | | | | | **NK cells** | **Monocytes** |
| --- | --- | --- | --- | --- | --- | --- | --- | --- | --- | --- |
| *Genes* | *Cd4* | *Cd79a* | *Cd79b* | *Ms4a1* | *Pax5* | *Ighd* | *Ighm* | *Il7r* | *Klrb1c* | *Irf8* |
| *Cd4* | 1.000 | 0.833 | 0.858 | 0.791 | 0.816 | 0.777 | 0.815 | 0.829 | 0.670 | 0.935 |
| *Cd79a* | 0.833 | 1.000 | 0.988 | 0.961 | 0.978 | 0.897 | 0.962 | 0.984 | 0.725 | 0.930 |
| *Cd79b* | 0.858 | 0.988 | 1.000 | 0.940 | 0.955 | 0.878 | 0.976 | 0.977 | 0.806 | 0.913 |
| *Ms4a1* | 0.791 | 0.961 | 0.940 | 1.000 | 0.916 | 0.973 | 0.916 | 0.942 | 0.679 | 0.914 |
| *Pax5* | 0.816 | 0.978 | 0.955 | 0.916 | 1.000 | 0.861 | 0.957 | 0.982 | 0.616 | 0.929 |
| *Klrb1c* | 0.670 | 0.725 | 0.806 | 0.679 | 0.616 | 0.611 | 0.738 | 0.712 | 1.000 | 0.595 |
| *Irf8* | 0.935 | 0.930 | 0.913 | 0.914 | 0.929 | 0.893 | 0.889 | 0.925 | 0.595 | 1.000 |
| *Ighd* | 0.777 | 0.897 | 0.878 | 0.973 | 0.861 | 1.000 | 0.870 | 0.905 | 0.611 | 0.893 |
| *Ighm* | 0.815 | 0.962 | 0.976 | 0.916 | 0.957 | 0.870 | 1.000 | 0.965 | 0.738 | 0.889 |
| *Il7r* | 0.829 | 0.984 | 0.977 | 0.942 | 0.982 | 0.905 | 0.965 | 1.000 | 0.712 | 0.925 |
